# Identification of proteins in laser-microdissected small cell numbers by SELDI-TOF and Tandem MS

**DOI:** 10.1186/1472-6750-4-30

**Published:** 2004-12-03

**Authors:** Grazyna Kwapiszewska, Markus Meyer, Ralf Bogumil, Rainer M Bohle, Werner Seeger, Norbert Weissmann, Ludger Fink

**Affiliations:** 1 Department of Pathology, Justus-Liebig University Giessen, Langhansstr.10, 35392 Giessen, Germany; 2 Ciphergen Biosystems GmbH, Göttingen, Germany; 3 Department of Internal Medicine II, Justus-Liebig University Giessen, 35392 Giessen, Germany

## Abstract

**Background:**

Laser microdissection allows precise isolation of specific cell types and compartments from complex tissues. To analyse proteins from small cell numbers, we combine laser-microdissection and manipulation (LMM) with mass spectrometry techniques.

**Results:**

Hemalaun stained mouse lung sections were used to isolate 500–2,000 cells, enough material for complex protein profiles by SELDI-TOF MS (surface enhanced laser desorption and ionization/time of flight mass spectrometry), employing different chromatographic ProteinChip^® ^Arrays. Initially, to establish the principle, we identified specific protein peaks from 20,000 laser-microdissected cells, combining column chromatography, SDS-PAGE, tryptic digestion, SELDI technology and Tandem MS/MS using a ProteinChip^® ^Tandem MS Interface. Secondly, our aim was to reduce the labour requirements of microdissecting several thousand cells. Therefore, we first defined target proteins in a few microdissected cells, then recovered in whole tissue section homogenates from the same lung and applied to these analytical techniques. Both approaches resulted in a successful identification of the selected peaks.

**Conclusion:**

Laser-microdissection may thus be combined with SELDI-TOF MS for generation of protein marker profiles in a cell-type- or compartment-specific manner in complex tissues, linked with mass fingerprinting and peptide sequencing by Tandem MS/MS for definite characterization.

## Background

Investigation of cell-type specific gene expression and regulation in complex tissues is hampered by the lack of accuracy of cell isolation and sensitivity of post-isolation analysis. Laser-microdissection techniques have proven to be a reliable tool for selectively harvesting cell clusters or single cell profiles from stained tissue sections for mRNA and protein investigation. When combined with qualitative and quantitative PCR, mRNA can be successfully analysed from a few cells [1-3]. The combination of laser-microdissection and cDNA arrays allows investigation of differential gene expression in a cell type specific manner for a multitude of genes in parallel [4,5]. For proteome analysis, two-dimensional polyacrylamide gel electrophoresis (2D-PAGE) has been previously performed from 50,000 to 250,000 microdissected cells, followed by peptide mass finger printing of single spots [6-8]. Isolation of such high cell numbers by laser-microdissection is extremely time consuming or even impracticable in complex tissues.

Recently, several groups have successfully combined laser-microdissection with surface-enhanced laser desorption/ionization mass spectrometry (SELDI MS) to generate reproducible MS profiles from 200–5,000 cells [9-14]. Changes in these protein profiles resulting from different biological conditions can be employed as biomarkers. Similarly, using matrix-assisted laser desorption/ionization mass spectrometry (MALDI MS), spectra could be generated from 500 to 2,500 microdissected cells [15,16]. However, the definite identification of peptides/proteins underlying single biomarkers from laser-microdissected material demands substantially higher cell amounts and laborious, time-intensive procedures. To date, only one biomarker has been identified after profiling of laser-microdissected tissue. In this case, Melle *et al*. combined 2D-PAGE with peptide mapping and tandem mass spectrometry to identify a protein significantly higher expressed in tumour tissue and confirmed the identity by immunodepletion assay and immunhistochemistry [13].

In addition to generating compartment-specific biomarker profiles, we aimed to develop alternative strategies circumventing the laborious 2D-PAGE for definite protein identification using limited cell numbers derived from microdissected material. The strategies were evaluated at septal and vascular compartments of the complex lung tissue.

## Results

### Generation of protein profiles by SELDI mass spectrometry

**Figure 1:**
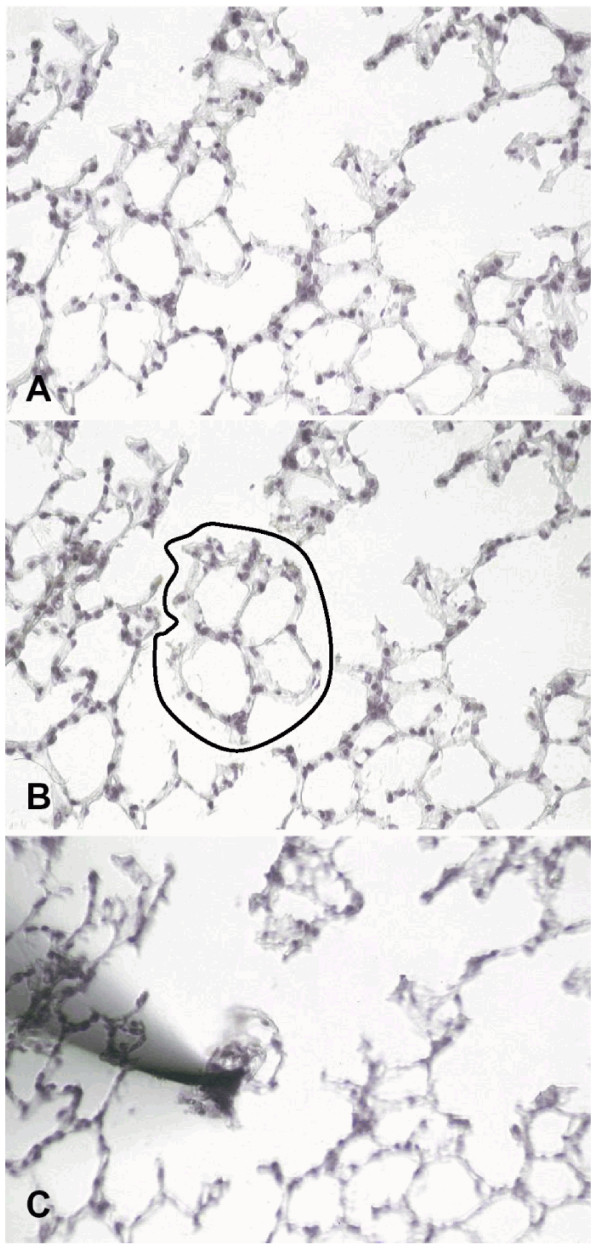
Isolation of an alveolar septum by laser-assisted microdissection and manipulation from a hemalaun stained frozen lung section (magnification 200×). A) Alveolar septum is selected for isolation. B) Laser photolysis is used to disconnect the cells from adjacent ones. C) Septum cells adhere tightly to the approximated sterile needle and can be transferred into a reaction tube.

Laser-microdissection and manipulation was used to isolate 500–2,000 alveolar septum cells (Figure 1), which were then transferred into 15 μl of HEPES/Triton X-100 lysis buffer. Approximately 30 intrapulmonary vessels (corresponding to 500–2,000 cell profiles) were microdissected from tissue sections and lysed identically. Following the first isolation, the remaining cell pellet was subjected to Urea/Thiourea/CHAPS (UTC) buffer.

**Figure 2:**
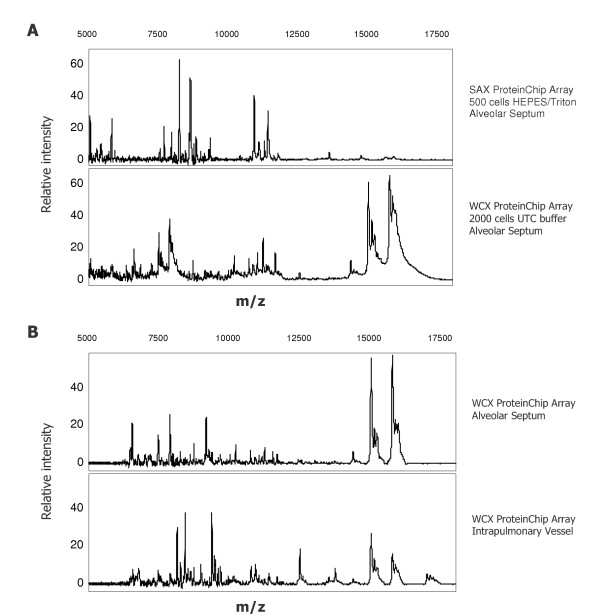
Protein profiling of laser-microdissected alveolar septum cells and intrapulmonary vessels for detection of differentially expressed proteins (m/z = mass/charge). A) SELDI-TOF spectra comparison of a SAX ProteinChip^® ^Array profile from HEPES/Triton X-100 lysed septum cells and WCX ProteinChip Array^® ^profile from septum cells treated with UTC buffer. B) Spectra comparison of intrapulmonary vessel profiles and alveolar septum (2,000 cells) after UTC lysis and binding onto WCX ProteinChip^® ^Array.

**Figure 3:**
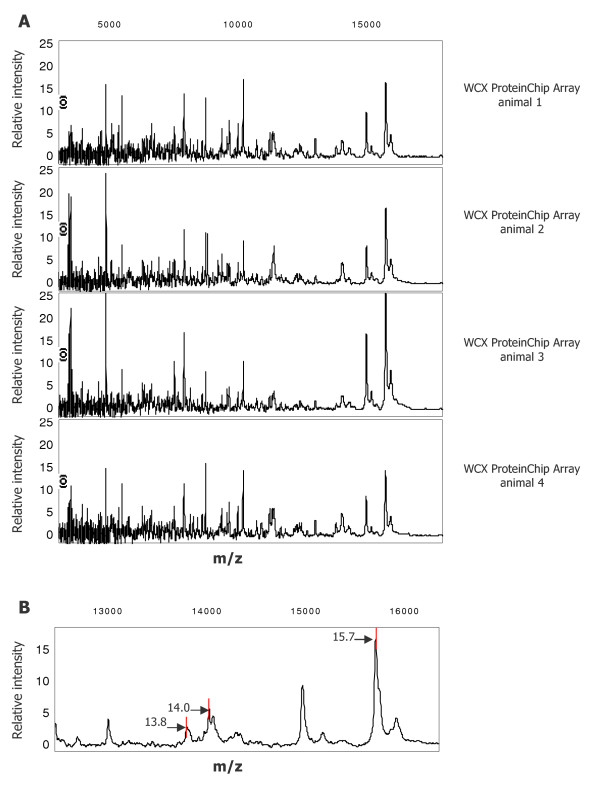
SELDI-TOF spectra of alveolar septum cells (2,000 cells) from four different mice A) Laser-microdissected alveolar septa from 4 different mice were lysed in UTC lysis buffer and profiled on a WCX ProteinChip^® ^Array. Spectra of the molecular range between 3–18 kD are depicted. B) Zoom of the region between 12.5–16.5 kD: A representative spectrum showing three peaks at 13.8, 14.0 and 15.7 kD which were selected for further biochemical identification.

a) To assess the effect of different lysis buffers and different surface properties of the ProteinChip^® ^Arrays, HEPES/Triton X-100 protein lysate as well as UTC lysate was applied independently to SAX (strong anionic exchanger) and WCX (weak cationic exchanger) ProteinChip^® ^Arrays. Compared to the weaker HEPES lysis, UTC buffer resulted in a remarkably higher yield of peaks (signal to noise ratio (S/N) ≥ 3) on WCX arrays. On the other hand, HEPES buffer gave more individual spectra on SAX arrays. 500 cells were sufficient to detect more than 35 peaks on both SAX and WCX arrays. However, immobilization of 2,000 cells resulted in over 50 peaks on WCX arrays (Figure 2A). In regard of limiting cell numbers a two step extraction procedure (HEPES buffer followed by UTC) was proven to be useful to display a higher amount of peaks for differential expression analysis. Therefore this procedure was used for all further profiling experiments.

b) Comparing alveolar septum cells to intrapulmonary vessels, the profiles differed considerably, with only few overlapping peaks. Representative profiles are given in Figure 2B.

c) Four representative SELDI TOF MS spectra of alveolar septum cells from four different animals are shown in Figure 3A. These data show good reproducibility of protein detection by SELDI-MS in agreement with previous studies by Zhukov *et al*. [14] who also assessed reproducibility of SELDI-MS after using laser-microdissected lung material. To determine the limit in protein abundance for further identification, peaks with different intensities were chosen: one high-abundant protein with molecular weight of 15.7 kD and two low-abundant proteins of 13.8 and 14.0 kD, respectively. The three protein peaks with different intensities are presented in the zoomed area of Figure 3B. For identification of these proteins following strategies were evaluated.

### Enrichment of proteins from microdissected cells by column chromatography and SDS-PAGE

**Figure 4:**
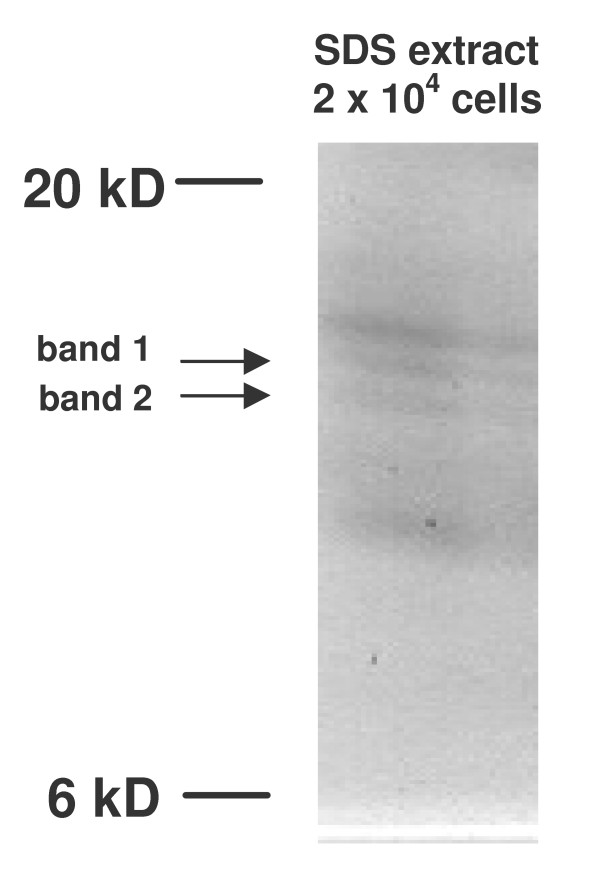
SDS extract of 20,000 microdissected alveolar septum cells separated by SDS-PAGE. After first extraction with UTC buffer a second extraction step with SDS sample buffer was performed. The amount equivalent to 20,000 cells of this extract was separated on a 15% SDS gel. Band 1 and band 2 correspond to the selected protein peaks of 13.8 and 14.0 kD from WCX Array experiments (Figure 2).

Protein lysate of 10,000 to 20,000 microdissected septum cells was extracted by UTC buffer, the remaining material of the same needles was further extracted by SDS sample buffer. Protein samples from UTC and SDS extracts were separated by SDS-PAGE. Although both extracts showed several colloidal Coomassie Brilliant Blue (CBB) stained bands the SDS extract revealed several proteins in the MW region between 12–16 kD (Figure 4). Some of the clearly separated gel bands in the molecular weight region of the target proteins were excised and subjected to in-gel Trypsin digestion. Band 1 represented the 14.0 kD peak while band 2 corresponded to the 13.8 kD peak as identified later by peptide mapping and MS/MS experiments.

The 15.7 kD protein was enriched by micro-spin column chromatography using a Q HyperD^® ^spin column (Ciphergen Biosystems, CA). The protein extract equivalent to approximately 50,000 cells was applied to the column and 6 fractions were eluted according to a stepwise pH gradient and concentrated by trichloroacetic acid precipitation. Aliquots of 3 μl (100 μl total volume) of each fraction were applied to a NP20 ProteinChip Array (hydrophilic chemistry) in order to detect the presence and enrichment of the selected proteins. In the organic fraction (last elution step) all three proteins (13.8, 14.0 and 15.7 kD) were detected on an NP20 ProteinChip. After separation of the complete organic fraction by SDS-PAGE, proteins were stained by colloidal CBB. A protein band with estimated molecular weight between 15–16 kD was excised and subjected to tryptic digestion (not shown).

### Identification of the isolated target proteins by tryptic peptide mass fingerprinting on ProteinChip^® ^Arrays and direct peptide fragmentation by Tandem MS/MS

**Figure 5:**
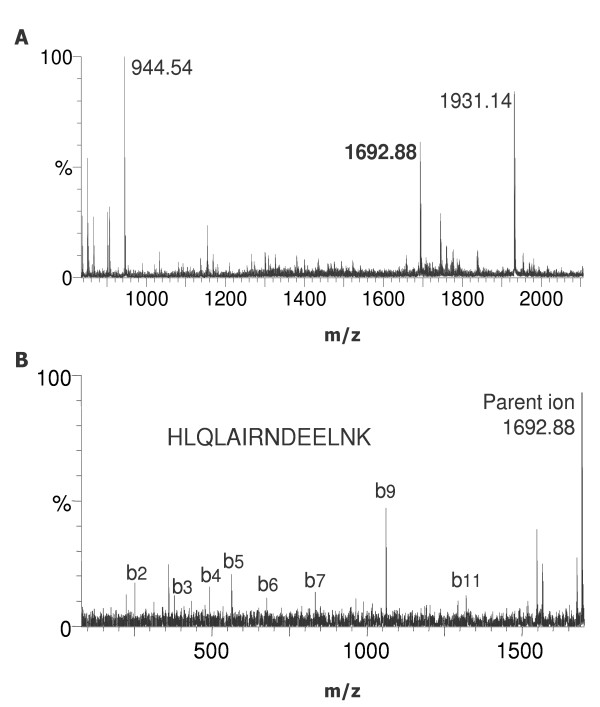
MS/MS analysis of the tryptic fragments of band 2. A) Tryptic fragments of band 2 were applied to NP20 ProteinChip^® ^Arrays and measured on a ProteinChip^® ^Interface coupled to a Tandem MS. The peptide of 1692.88 D was selected for MS/MS analysis. B) CID-MS/MS spectrum from tryptic fragment with m/z 1692.88.

Protein bands isolated from the gel were subjected to Trypsin digestion. Gel pieces were extracted twice and the resulting peptide fragments applied to NP20 and H4 ProteinChip^® ^Arrays with hydrophilic and hydrophobic chromatographic properties, respectively. Peptide mass fingerprinting of the gel bands was done using the PBSIIc instrument. The results are given in Table 1. Two histone proteins (band 1 and 2) and haemoglobin beta were the first candidates in Profound database search. For unambiguous identification, selected peptides were sequenced directly from the arrays by collision-induced dissociation (CID), using a ProteinChip^® ^Interface coupled to a Tandem mass spectrometer [17-19]. Representative MS and MS/MS spectra from band 2 are given in Figure 5. The peptide with an m/z ratio of 1692.88 (Figure 5A) was selected for sequencing by CID-MS/MS (Figure 5B). The obtained sequence was assigned to histone proteins (H2A1 or H2A4, Table 1). It is notable that the molecular masses of the identified proteins correlated well with the results obtained from the profiling experiments (Table 1 and Figure 3B). Analysis of tryptic peptide fragments from band 3 confirmed the molecular mass from profiling experiments (15.7 kD) and showed strong evidence for haemoglobin beta (Sequence coverage 45.7%).

### Enrichment of the target protein markers from tissue sections by SDS-PAGE

**Figure 6:**
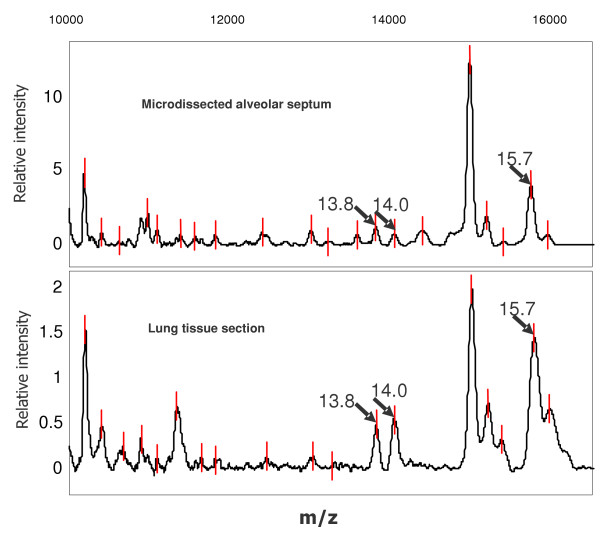
SELDI-TOF spectra comparison of profiles from microdissected cells versus lung tissue sections. Microdissected alveolar septum cells as well as five lung tissue sections were lysed in UTC buffer and bound onto WCX ProteinChip^® ^Arrays. All three investigated target proteins could be recovered from the tissue section derived profile as well.

Due to the labour-intensive requirements of isolating high cell numbers by laser-microdissection, we sought to recover the target proteins in whole lung tissue sections, intending to use this material for subsequent protein identification. Between 5–7 cryosections of lung tissue (10 μm) previously known to contain the target proteins from laser-microdissection were collected and proteins were isolated following the procedure as for the laser-microdissected cells. Applying an aliquot of approximately 4,000 cells to a spot of WCX ProteinChip^® ^Array, we were able to recover the target protein markers within the homogenate spectrum (Figure 6). Using another aliquot of the section material for SDS-PAGE, we isolated single bands of expected weight, as already described for the microdissected cells. This material was subjected to tryptic digestion and peptide mass fingerprint. Again, the two histone proteins and haemoglobin beta were identified.

## Discussion

The combination of laser-microdissection and mass spectrometry has been shown to be a reliable tool for compartment and cell type-specific biomarker profiling in complex tissues. Several groups have described the successful generation of mass spectra from as few as 500 to 2,000 cells after microdissection [11,15,16] which was reproduced in the present investigation. Such spectra may be employed to ascertain the cellular origin of microdissected samples. Moreover, when showing differential expression under various biological conditions, mass spectral peaks may serve as biomarkers, independent of their identification. This fast and convenient technique thus represents a valuable tool to provide disease- or status-specific protein marker patterns to be used for diagnostic and predictive purposes [20-22]. In the present investigation we could confirm feasibility and excellent reproducibility of this approach when analyzing lung tissue compartments.

To investigate proteins on 2D-gels, high amounts of cells are required. Therefore, using this approach as a starting point for protein profiling with subsequent MS identification, 50,000 to 250,000 microdissected cells have to be introduced per gel. Our aim was to minimize this laborious procedure, being hardly compatible with microdissection of minor cellular compartments. Thus, we first generated compartment specific profiles from laser-microdissected material by mass spectrometry and subsequently collected high cell numbers to identify the previously selected proteins. Moreover, using an interface to a Tandem MS/MS instrument, analysis of tryptic mass fingerprints can be performed from the same array without need for additional material. To reduce the demand of material on the gel, different staining techniques can be used (e.g. silver or SYPRO RUBY^® ^fluorescence staining). While advantageous in gel staining due to higher sensitivity, the problems are shifted towards MS technique: the identification may fail due to minute amounts of protein per gel spot. Therefore, in our study we performed robust and easy CBB staining to be able to detect the required amount of material in MS techniques. Additionally, for low abundant proteins, direct Trypsin digestion of an eluted, gel-resolved protein can be performed directly on the array. Marker protein isolation and enrichment of the protein peak may also be enhanced by application of suitable pH and salt conditions directly on the array.

A promising alternative for labour-intensive 2D-gel applications can be the detection of biomarker proteins from microdissected cells and their subsequent identification in tissue slices. Since the exact weight of the target protein is known from preceding experiments with microdissected cells, the section homogenate can be screened for the respective peaks. As tissue sections are easy and fast to obtain, they were used to generate profiles on WCX arrays from the same lungs as from microdissection. While these spectra differed partially from those derived from microdissected alveolar septum cells, the peaks corresponding to the pre-defined target proteins were easily detected in the homogenate spectrum. In addition, corresponding bands could be detected by SDS-PAGE and subsequent tryptic mass fingerprinting confirmed the identity of histones H2B F and H2A1 and haemoglobin beta.

Typically, high-resolution 2D techniques require several days from sample application to the final staining of protein spots. Isolation of 500 to 2,000 cells by laser-microdissection requires minutes to hours, depending on the targeted cell type, tissue area or organ compartment. Array pre-treatment, immobilization of the lysate and washing lasts around 90 minutes and MS measurement is performed within few minutes. This calculation may reveal the time saved by our approach.

Due to the accuracy of measurement we found that the molecular weight derived from SELDI mass spectrometry corresponded very well with the exact protein mass. For the three investigated proteins, the mass accuracy was approximately 0.1% for external calibration and is thus remarkably higher than using 2D-PAGE. Nevertheless, the exact molecular weight alone is insufficient to identify the concerning protein directly via database search.

## Conclusions

Combination of laser-microdissection with SELDI-TOF MS generates reproducible and credible biomarker profiles in a cell-type- or compartment-specific manner from complex tissues. For identification of underlying peptides/proteins, this approach may be combined with enrichment and isolation strategies, linked with mass fingerprinting by SELDI-TOF MS and peptide sequencing by Tandem MS/MS for definite chemical characterization. These techniques allow analysis of differential protein expression of low cell numbers microdissected from complex tissues.

## Methods

### Mouse lung preparation

All animal experiments were approved by local authorities [Regierungspräsidium Giessen, no. II25.3-19c20-15(1) GI20/10-Nr.22/2000]. Lung preparation was performed as described previously [5]. In brief, male BALB/c mice (Charles River, Sulzfeld, Germany, 20–22 g) were exposed to normobaric normoxia in a chamber at FiO_2 _= 0.21. After 1 day, animals were sacrificed; lungs were flushed via the pulmonary artery, and 800 μl prewarmed TissueTek^® ^(Sakura Finetek, Zoeterwoude, The Netherlands) were instilled into the airways via a tracheal cannula. Afterwards, lungs were excised and frozen in liquid nitrogen immediately.

### Laser-assisted microdissection

Laser-microdissection and manipulation was performed as described previously [1-3,5]. In brief, cryosections (10 μm) from lung tissue were mounted on glass slides. After hemalaun staining for 30 sec, sections were subsequently immersed in 70% and 96% ethanol and stored in 100% ethanol until use. Not more than 10 sections were prepared at the same time to restrict storage time. Alveolar septum cells and intrapulmonary vessels, respectively, were microdissected under visual control using the Laser Microbeam System (P.A.L.M., Bernried, Germany) and isolated by a sterile 30 G needle (Figure 1). Needles with adherent material were transferred into a reaction tube containing HEPES/Triton X-100 lysis buffer (50 mM HEPES, pH 7.2, 1% Triton X-100).

### Comparative protein expression profiling by SELDI

Needles with adherent cells were transferred to the reaction tubes containing 15 μl of the HEPES/Triton X-100 lysis buffer. After vigorous shaking for 30 min at room temperature, samples were centrifuged at 14,000 g for 10 min. 3 μl of this supernatant were directly applied to the spots of SAX and WCX ProteinChip^® ^Arrays and incubated in a humid chamber for 1 h. For profiling, SAX ProteinChip^® ^Arrays were preincubated in SAX Binding Buffer (100 mM Tris, pH 8.5, 0.02% Triton X-100).

After first extraction of the microdissected material using HEPES buffer, 15 μl of UTC buffer (6 M Urea, 2 M Thiourea, 2% CHAPS, 75 mM DTT) were applied to needles/cells. The pellet in UTC buffer was centrifuged for 10 min at 14,000 g and the complete supernatant was used for profiling on WCX ProteinChip^® ^Arrays. These were pre-treated with 10 mM HCl for 5 min and equilibrated two times with WCX binding buffer (100 mM NaOAc, pH 4.5, 0.02% Triton X-100) for 5 min. Fifteen μl of UTC supernatant were diluted 1:10 v/v in WCX binding buffer and incubated under vigorous shaking for 45 min. In order to deal with high amounts of volume (150 μl) the Bioprocessor (Ciphergen Biosystems. Inc.) was applied. After removal of the samples every spot was washed twice using binding buffer followed by a final 10 sec water rinse. After air-drying, saturated sinapinic acid (0.6 μl) dissolved in 50% acetonitrile and 0.5% trifluoracetic acid (TFA) was added twice. Subsequently, mass analysis of bound peptides/proteins was performed using the Ciphergen PBS IIc system. ProteinChip^® ^Arrays were analyzed by averaging 100–150 laser shots collected in the positive mode. Optimization range of the time lag focusing was set between 10–30 kD. Deflector settings were used to filter out peaks with <2000 *m/z*. Calibration was performed externally using purified peptide and protein standards. Obtained spectra were analyzed by the ProteinChip Software version 3.01.

### Small scale column chromatography and SDS-PAGE

SDS-PAGE was performed as described previously [23] with minor modifications. We used 15% acrylamid separation and 5% stacking gels in a mini-gel chamber (Roth, Karlsruhe). Supernatants of UTC buffer extraction containing approximately 2000 cells/μl were mixed 1:1 with twofold concentrated sample buffer (100 mM Tris-Cl pH 6.8, 4% SDS, 20% Glycerin, 3% DTT, 0.05% Bromphenolblue). Samples were boiled for 3 min and a total volume of 20 μl (equivalent to 20,000 cells) was loaded onto single lanes of the gel. Afterwards, 1 μl SDS sample buffer per 1,000 cells was added to the needles in order to solubilize remaining proteins after UTC buffer extraction. SDS extracts were also applied to SDS gels.

For selective enrichment of marker proteins we used small sized anionic exchange spin columns resulting in 6 fractions after elution using a stepwise pH gradient from pH 9–3 and an organic fraction. The protein amount reflecting 50,000 cells was loaded onto a Q HyperD spin column and aliquots of the eluted fractions were analyzed on NP20 ProteinChip^® ^Arrays to reveal the enrichment of selected protein peaks in a certain fraction. The fractions containing the enriched proteins were concentrated by TCA precipitation in order to apply the complete fraction to a single lane of an SDS gel. Staining was performed by colloidal Coomassie brilliant blue (CBB, Roth).

### Protein tryptic digestion (peptide mass fingerprint)

The CBB stained bands matching the expected molecular weight regions of selected proteins were excised and subjected to Trypsin digestion. Gel pieces were washed three times with 400 μl of 100 mM ammonium bicarbonate and 50% acetonitrile for 15 min followed by 15 min incubation in 100% acetonitrile. After removal, gel pieces were dried shortly in a speed-vac centrifuge. Depending on the gel volume, 10–15 μl of a Trypsin solution (20 ng Trypsin/μl in 25 mM ammonium bicarbonate) was applied and digestion was performed overnight (16 h) at 37°C. Afterwards, reaction tubes were centrifuged and 0.5–1.5 μl aliquots of each supernatant were applied to the spots of H4 (hydrophobic surface coating) and NP20 (hydrophilic surface coating) ProteinChip^® ^Arrays. A 20% matrix solution of alpha-4 hydroxy-cinnamic acid (CHCA) was applied to the spots. The remaining gel pieces were extracted with a 60% acetonitrile and 0.2% TFA solution for 1 h with a 5 min sonication to extract remaining organic peptides. Supernatants from this second extraction step were also applied to H4 and NP20 ProteinChip^® ^Arrays. All Arrays were measured in the PBS IIc system by averaging 150 laser shots. After subtraction of all peaks also present in the blank gel piece (e.g. Trypsin autolysis peaks), m/z values were submitted to Profound and Mascot for database searching.

### Tandem MS/MS analysis

Peptides from tryptic digestion were applied to NP20 ProteinChip^® ^Arrays and 2 × 0.6 μl of a saturated CHCA solution was added. For quality control of the peak intensities, the NP20 Arrays were analyzed in a PBS IIc instrument. Afterward, the arrays were transferred to a Tandem MS instrument. Data were acquired on a Micromass QTOF II (Manchester, UK) tandem quadrupole-time of flight (Q-TOF) mass spectrometer equipped with a PCI 1000 ProteinChip^® ^Tandem MS Interface (Ciphergen Biosystems). Ions were created using a pulsed nitrogen laser operating with 30 pulses/sec. Nitrogen gas was used for collisional cooling of formed ions and argon gas was used for all low-energy collision-induced dissociation experiments. The system was externally calibrated in MS/MS mode using the parent ion and selected fragments of adrenocorticotropic hormone (ACTH) human fragment 18–39 (m/z = 2465.1983; Sigma Aldrich).

## Authors' contributions

GK: laser-microdissection, preparation of samples, PAGE, SELDI-MS

MM: SELDI-MS and optimalisation for microdissected material

RB: tandem MS measurements

RMB: instruction to laser-microdissection

WS: design of project, preparation of the manuscript

NW: animal model, preparation of the lungs

LF: design of project, preparation of the manuscript

## Figures and Tables

**Table 1:** Protein database query of the three investigated target protein bands. Details and specifications of the analysis that resulted in identification of the biomarker proteins are given. MW: molecular weight

**Gelband No.**	**MW calc.**	**Sequence coverage %**	**Score profound**	**MS/MS sequence**	**Acces. No. Swissprot**	**Protein name**
band 1	13.797	63	2.24	**AMGIMNSFVNDIFER**	P10853	Histone H2B F
band 2	14.042	31		**HLQLAIRNDEELNK**	P22752	Histone H2A.1
	14.522				P10812	H2A.4
band 3	15.708	45.9	1.17		P02088	Haemoglobin β
